# Multi-scale convolutional recurrent neural network for psychiatric disorder identification in resting-state EEG

**DOI:** 10.3389/fpsyt.2023.1202049

**Published:** 2023-06-27

**Authors:** Weizheng Yan, Linzhen Yu, Dandan Liu, Jing Sui, Vince D. Calhoun, Zheng Lin

**Affiliations:** ^1^Department of Psychiatry, Second Affiliated Hospital, Zhejiang University School of Medicine, Hangzhou, China; ^2^Tri-Institutional Center for Translational Research in Neuroimaging and Data Science (TReNDS), Georgia State University, Georgia Institute of Technology, Emory University, Atlanta, GA, United States

**Keywords:** deep learning, EEG, MCRNN, psychiatric disorder, biomarker

## Abstract

**Background:**

Accurate classification based on affordable objective neuroimaging biomarkers are important steps toward designing individualized treatment.

**Methods:**

In this work, we investigated a deep learning classification model, multi-scale convolutional recurrent neural network (MCRNN), to explore psychiatric disorder-related biomarkers by leveraging the spatiotemporal information of resting-state EEG (rsEEG) using a multiple psychiatric disorder database containing 327 individuals diagnosed with schizophrenia, bipolar, major depressive disorders, and healthy controls. All subjects were mapped to a shared low-dimensional subspace for intuitively interpreting the inter-relationship and separation of psychiatric disorders.

**Results:**

Psychiatric disorders were identified using rsEEG with high accuracy ranged from 78.6 to 91.3% in patient vs. controls two-class classification, and 68.2% in four-class classification. The control-to-schizophrenia trajectory interpretated by the model was consistent with the disease severity in clinical observation.

**Conclusion:**

The MsRNN demonstrated a capability in extracting discriminative rsEEG biomarkers for psychiatric disorder classification, indicating its potential to facilitate our understanding of psychiatric disorders and monitoring interventions.

## Introduction

Major psychiatric disorders, including major depressive disorder (MDD), bipolar disorder (BP), and schizophrenia (SZ), are among the most severe and impactful mental illnesses. They lead to decreased quality of life, premature death/disability in many patients, and increased health care costs. Major psychiatric disorders are highly comorbid in specific symptoms but are heterogenous in underlying mechanisms, causing difficulties in diagnosis and treatment (1, 2). Current psychiatric diagnosis guidelines are based on phenomenological descriptions, with no assay-based biological criteria underlying diagnosis (3). For instance, due to the overlapping clinical symptoms at onset, about 60% of BP patients are initially diagnosed as MDD and have to wait 5–10 years before receiving appropriate diagnoses (4). The biological heterogeneity of psychiatric disorders has a substantial effect on treatment response, resulting in the unpredictability of therapeutic effects. For example, the treatment response of treatment-resistant major depression using transcranial magnetic stimulation (TMS) varies from 45 to 60% (5). Therefore, it is urgent to discover biomarkers that can be used for accurate disorder diagnosis.

Non-invasive neuroimaging techniques, such as structural magnetic resonance imaging (sMRI) (6), resting-state functional MRI (7–9), or rsEEG (10, 11), has been widely studied for identifying disorder-related biomarkers or subtypes. In comparison to MRI, EEG is an inexpensive measurement with higher time resolution and non-magnetic effects, making it a viable healthcare tool in a variety of clinical environments. Previous work (10) identified two clinically relevant subtypes of post-traumatic stress disorder and MDD based on functional connectivity patterns in EEG. Decades of studies in EEG provide us with a variety of metrics [e.g., coherence (12), phase synchronization (13), and phase-slope index (14)] to quantify neural interactions as well as interpreting discoveries (15). However, few of the studies directly extracted spatiotemporal representations from EEG time series (16). Compared to the standard machine learning method, deep learning can encode more robust discriminative neuroimaging representations by characterizing potentially non-linear high-level patterns existing in the input features (17). Our prior fMRI studies have demonstrated the effectiveness of deep learning in utilizing spatiotemporal information directly from fMRI time sequences (18, 19). Different from the frequency-based EEG methods reported by Wu et al. (20), which separated EEG into predefined frequency bands, the convolutional recurrent neural network can extract the weighted combinations of EEG channels and then process sequential information for more accurate classification.

This study aimed to investigate the feasibility of extracting rsEEG-based biomarker for major psychiatric disorder classification using deep learning models. In this study, we used an EEG-based deep learning method, multi-scale convolutional recurrent neural network (MCRNN), to leverage the spatiotemporal information of rsEEG for multi-disease classification and subtype discovery. By learning rsEEG time series from three major psychiatric disorders, the MCRNN can effectively learn nonlinear discriminative feature representations which inhibiting the disorder-unrelated covariates by mapping the EEG features into the disorder-specific subspace. In addition, the MCRNN uses various convolutional filters of different scales to extract spatial features from time series. The extracted features are concatenated, pooled, and then sent to the Gated Recurrent Unit (GRU) module, and finally passes through a fully connected layer and a Softmax layer to obtain categorical output.

## Materials and methods

### Overview of the study

**Figure 1:**
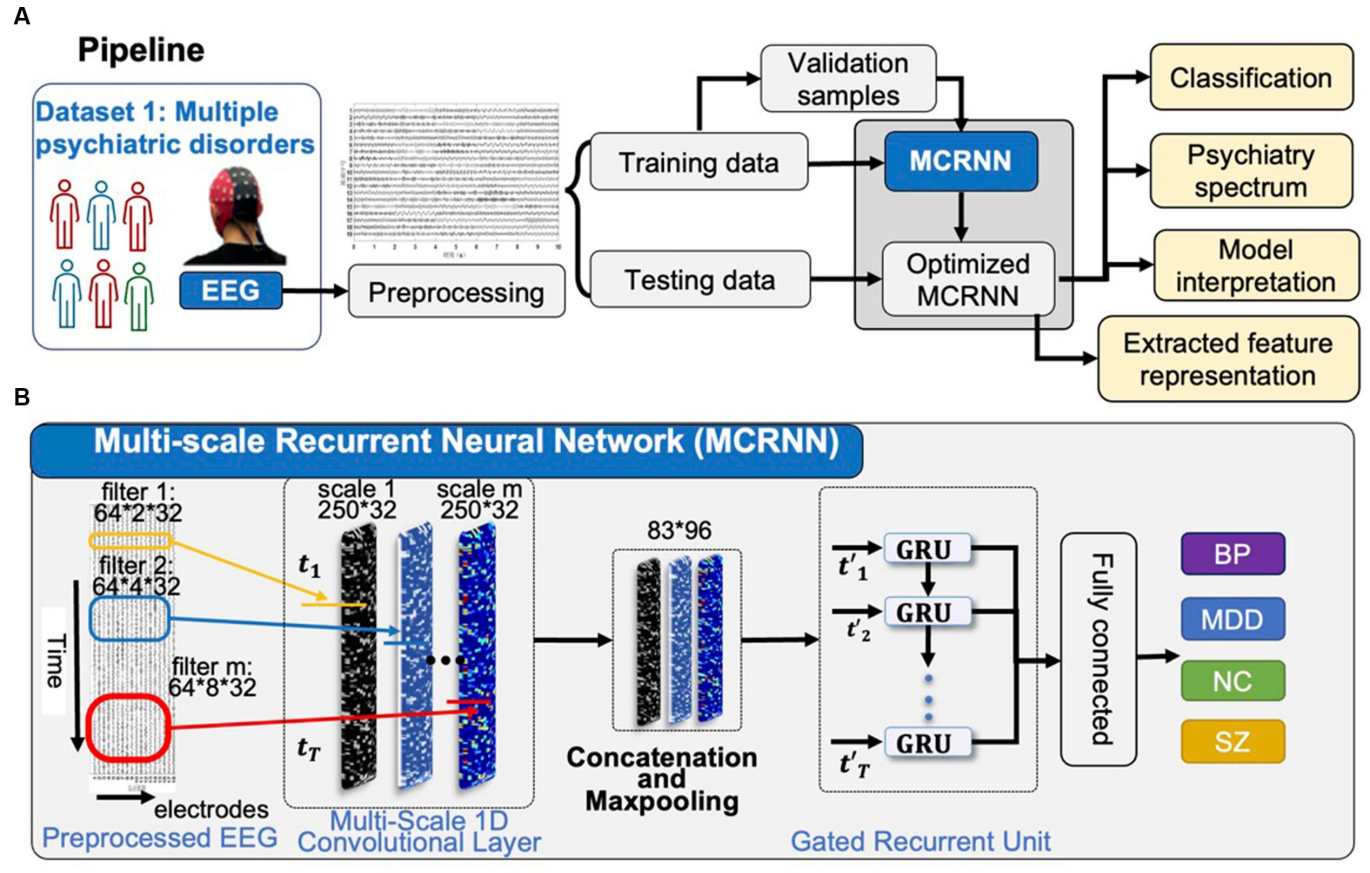
Overview of the study. **(A)** After preprocessing, EEG samples in Dataset are used for optimizing the parameters of MCRNN. The leave-one-out strategy is used for evaluating the model performance. The severity continuum of multiple psychiatric disorders is visualized. The model interpretation is applied for discovering the most discriminative EEG channels. **(B)** Details of the MCRNN model. The model has two main modules: multi-scale 1D convolutional layers as filters to map the preprocessed EEG sampling into various feature spaces, GRU for aggregating sequential information. MCRNN, multi-scale recurrent neural network; TMS, transcranial magnetic stimulation; HDRS, Hamilton depression rating scale; and GRU, gated recurrent unit.

As shown in Figure 1A, the preprocessed four-class rsEEG samplings were sent to MCRNN for model optimization. Leave-one-out cross-validation strategy was applied for evaluating the classification performance. The severity continuum of psychiatric disorders was visualized based on the high-level feature learning by MCRNN. The occlusion strategy was applied for explaining the discriminative power of each EEG channel.

### Participants

According to the Diagnostic and Statistical Manual of Mental Disorders (DSM-5), a total of 327 participants (BP = 72, MDD = 138, SZ = 70, NC = 47) were diagnosed after the semi-structured clinical interview by experienced psychiatrists. The demographic and clinical information of the participants were showed in Table 1. The participants were within the 16–40 age range, right-handed. Participants were excluded from enrollment if they had a currently substance abuse disorder, brain injury, a history of seizures, unstable medical condition, current pregnancy, or prior electroconvulsive therapy. Healthy controls were recruited from the local community after a semi-structured clinical interview to exclude any current or lifetime psychiatric disorders. All participants in this study signed written informed consent, which was approved by the ethics committee of the institutional review board of Second Affiliated Hospital, Zhejiang University School of Medicine.

**Table 1:** Demographic and clinical information of the participants.

	BP	MDD	SZ	NC	Statistic value
Gender (M/F)	25/47	47/91	28/42	22/25	*X*^2^ = 2.860, *p* = 0.414
Age (SD)	27.1 (7.7)	23.5 (8.2)	25.5 (7.5)	24.9 (3.8)	*F* (3, 323) = 2.163, *p* = 0.092
BRMS (SD)	9.28 (2.25)	-	-	-	
HDRS (SD)	-	25.56 (5.17)	-	-	
PANSS (SD)	-	-	73.7 (12.5)	-	

BP, bipolar disorder; MDD, major depressive disorder; SZ, schizophrenia; NC, normal control; BRMS, Bech-Rafaelsen Mania rating scale; HDRS, Hamilton depression rating scale; and PANSS, positive and negative syndrome scale.

### EEG data acquisition and preprocessing

The rsEEG was recorded at the first visit before accepting any neuromodulation therapy. Subjects were asked to sit on a chair comfortably in a quiet room, with eyes closed while staying awake. EEG was recorded using a wired Waveguard cap containing 64 Ag/AgCl recording channels (ANT Neuro, Hengelo, Netherlands). EEG electrodes were located following the 10/20 international placement system. Signals were sampled at 1 kHz, impedance were below 20 kΩ, referenced relative to CPz, online grounded at AFz, and amplified with an eegoTM amplifier (ANT Neuro, Hengelo, Netherlands). The electrodes placed at the supra-orbitally to the left eye were the bipolar recordings of electro-ocular activity (EOG). Resting-state EEG was continuously recorded for over 5 min for each participant.

The resting-state EEG data were processed using EEGLAB^1^ for further MCRNN analysis. The EEG data of each subject was processed as follow: downsampling to 250 Hz; band-pass 0.5–70 Hz; 260–300 1-s epochs were extracted after artifacts correction. Each epoch is represented with a 64(channel)×250(samplings) matrix.

### Multi-scale convolutional recurrent neural network

**Figure 2:**
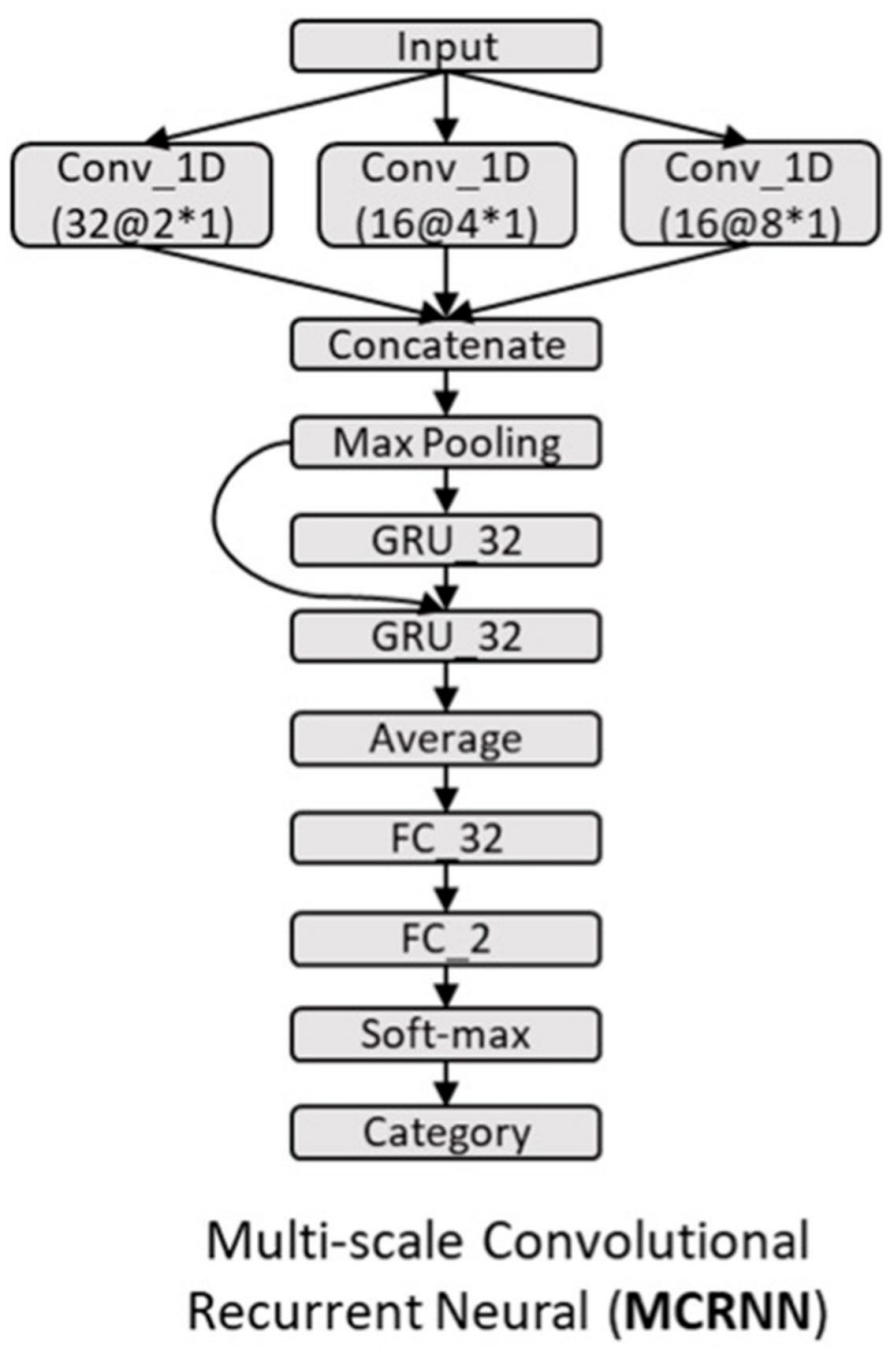
Architecture of MCRNN model. GRU_32, gated recurrent unit with 32 hidden nodes; Average, the average of the outputs of all GRU steps is connected to the next layer; FC_32, full-connected layer with 32 hidden nodes; Conv_1D, 1D convolutional layer; and a@b*c, the number of kernels is a, the size of kernel is b*c.

As shown in Figure 1B, multi-scale convolutional recurrent neural network (MCRNN) consisted of multiple 1D convolutional filters with different scales, one concatenation layer, one max-pooling layer, a gated recurrent unit (GRU), and an averaged layer for integrating the spatiotemporal information for classification. The preprocessed EEG signals were fed into the MCRNN model for parameter optimization. After optimizing the parameters, the model was saved for performance evaluation. The detailed architecture and mechanisms of the MCRNN were as follows: Multi-scale 1D convolutional layer expanded simple convolutional layers by including multiple filters of varying sizes in each Conv1D layer. The filter lengths used in the Conv1D were drawn from a logarithmic instead of a linear scale, leading to exponentially varying filter lengths (2, 4, and 8). Therefore, the dimensions of three different scales of convolutional filters are 64 (EEG channels) × 2 (filter length) × 32 (number of filters), 64 × 4 × 32, and 64 × 8 × 32. A concatenation layer then concatenated the incoming features, resulting in feature maps with size of 250 (time points) × 96 (feature dimension). Whereafter, a max-pooling layer performed down-sampling operation along the time dimensions with filter size 3, resulting in features whose size is 83(timepoints) × 96(feature dimension). The down-sampled features were the input of the subsequent GRU layers. As for the GRU layer, the size of the GRU’s hidden state was set to 32. The GRU layer could extract the sequential information and hidden states of the EEG signals. The extracted hidden states were then sent to the average-pooling layer. The fully connected layers and SoftMax were finally applied to get the final prediction results. The details of the model architecture are shown in Figure 2. More detailed discussions about the model architecture and hyper-parameter effects can be found in our previous work (18, 19).

The MCRNN model was trained by minimizing the cross-entropy loss using the Adam optimizer. The training batch size was set to 512. The learning rate started from 0.001 and decayed after each epoch with the decay rate of 0.01. To improve the generalization performance of the model and overcome overfitting, dropout (dropout rate: 0.5 for the convolutional layers, 0.3 for GRU module, 0.5 for fully connected layers), and L1,2-norm regularization (GRU kernel regular L1 = 10^−4^, L2 = 10^−4^) were also applied for regulating the model parameters. The validation samples were randomly generated from 20% of the preserved training samples. The training process was stopped when the validation loss stopped decreasing for 50 continuous epochs or when the maximum epochs (1,000 epochs) had been executed. The intermediate model which achieved the highest accuracy on the validation dataset was reserved for testing. The MCRNN was implemented using TensorFlow.^2^

## Results

### Multiple-psychiatric disorder classification results and psychiatric spectrum visualization

**Figure 3:**
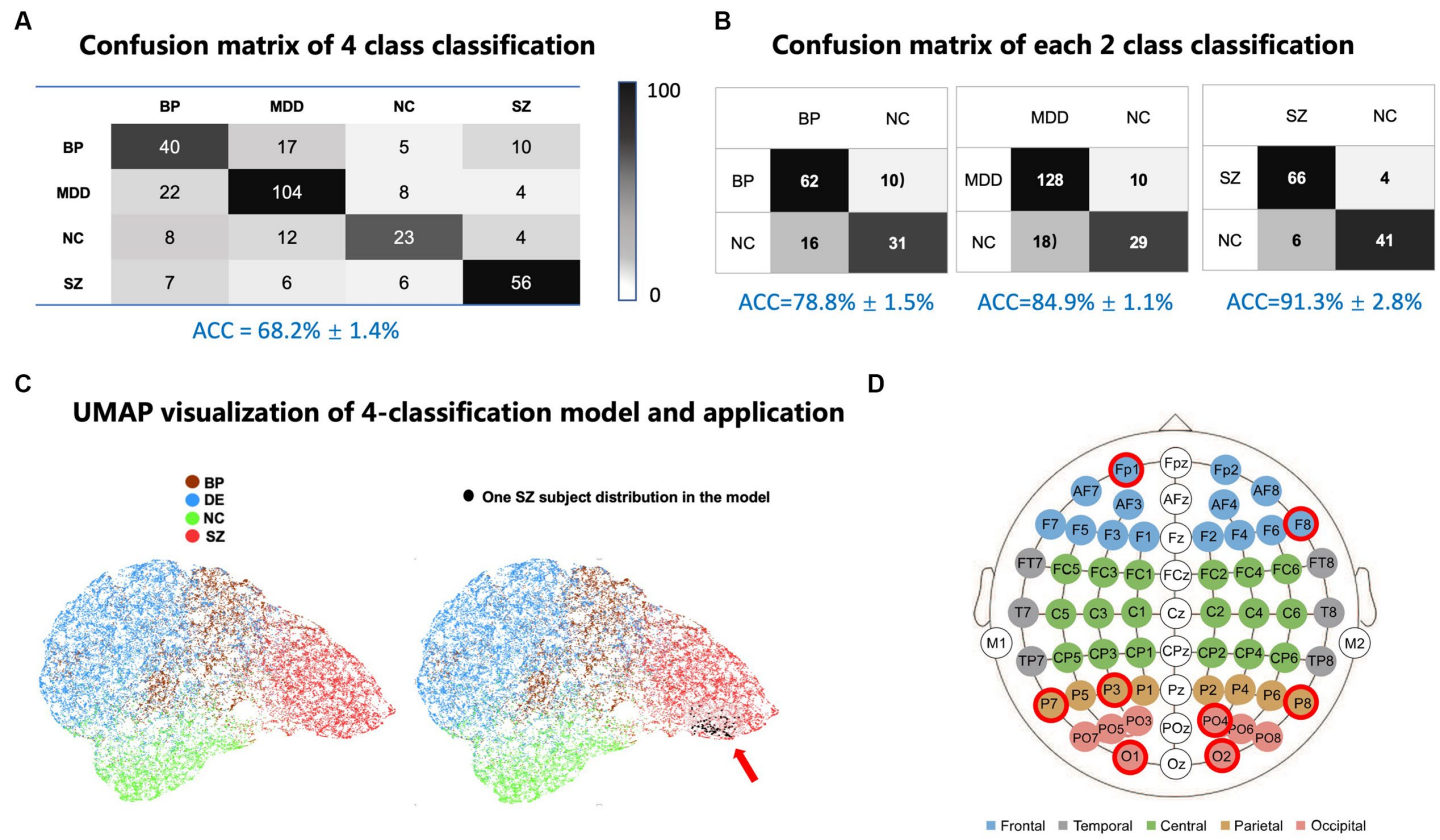
Classification results and model interpretation. **(A)** Confusion matrix of four-class classification results. **(B)** Confusion matrix of each two-class classification. **(C)** UMAP visualization of four-classification model and application, left: UMAP visualization of four-classification model; right: One SZ patient’s UMAP location (black dots) in four-classification model. Each dot represents an EEG epoch. **(D)** MCRNN four-class model interpretation. The red-circled are the most discriminative channels in the four-class classification task. UMAP, uniform manifold approximation and projection; MDD, major depressive disorder.

Dataset, which consists of 327 subjects, was used for training the MCRNN model. Leave-one-out strategy was applied for evaluating the classification performance. The accuracy of the four-class classification achieved 68.2% (Figure 3A). The accuracy of two-classification is from 78.6 to 91.3% (BP vs. NC = 78.6%, MDD vs. NC = 84.9%, and SZ vs. NC = 91.3%; Figure 3B). The confusion matrix showed that MDD and BP exhibit more overlaps than other psychiatric disorders. To visualize the severity continuum of various mental disorders, the 32-dimensional feature vectors for each EEG epoch, which were extracted from the second-last layer of MCRNN were mapped to a 2D plane using Uniform Manifold Approximation and Projection (UMAP). Figure 3C showed that the NC were closer to MDD and BP than to SZ. The severity “spectrum” also showed that MDD and BP were spatially close to each other. By combining with the Positive and Negative Syndrome Score (PANSS) information, we found that the “spectrum” discovered by MCRNN was also associated with the disease severity within the schizophrenia group (Supplementary Figure S1).

### Discriminative features discovery

All one-second-length EEG epochs of 327 subjects were utilized for optimizing the parameters of MCRNN. After optimizing the trained model, a feature discriminative analysis as mentioned above was done for capture the distinct contribution of each channel in classification. As shown in Figure 3D, the most discriminative channels were located at occipital lobe (O1, O2, and PO4), parietal lobe (P3, P7, and P8), and frontal lobe (Fp1, F8). Detailed information of the EEG electrodes can be found in Supplementary Table S1.

## Discussion

Due to the heterogeneity and comorbidity in neurobiological abnormalities in the clinical definitions of psychiatric disorders, discovering objective psychiatric biomarkers is imperative for understanding the pathophysiology and improving treatment. In this study, for the first time, we applied a deep learning-based model, MCRNN, to rsEEG for psychiatric-disorder classification and biomarker discovery. Classification accuracy ranged from 78.6 to 91.3% was achieved for the two-class classification, and 68.2% accuracy in four-class classification task, demonstrating the effectiveness of deep learning in extracting nonlinear discriminative information from rsEEG. In addition, this research utilized UMAP to visualize the spectrum of mental disorders based on the extracted EEG biomarkers.

Diagnosis criteria based on the clinical symptoms is not easy to be quantified. Mental disorders with similar symptoms have different underlying mechanisms. Given the circumstances, deep learning is a viable solution due to its capability in capturing high-level nonlinear discriminative features. In comparison to conventional time-frequency-based analysis, which manually extracts different frequency bands of rsEEG to generate functional connectivity features, the convolutional module of the MCRNN automatically learned spatial filters to map the original EEG into subspaces; the recurrent module subsequently integrated the sequential information for accurate classification. The four-class results demonstrated that the MCRNN could efficiently capture the discriminative neural activity patterns among psychiatric mental disorders such as MDD, BP, and SZ. The four-class confusion matrix and UMAP visualization results showed that BP and MDD had more overlaps than with other disorders. This phenomenon occurs because BP and MDD had many overlaps in clinical symptoms, and the core symptom of MDD can also be found in the depressive or mixed states of BP.

Deep learning-based approaches have the potential to facilitate psychiatric disorder diagnosis and comorbidity interpretation for two reasons: (1) deep learning architecture is quite flexible and suitable for various feature dimensions. Second, due to the gradient-descent and error back-propagation optimizing strategy, and (2) deep learning algorithms can automatically learn the manifold from the training data, which has been proved the right projection to the disease-related subspace (17, 21).

The ideal biomarkers should be both sensitive and specific for identifying mental disorders. By interpreting the deep learning model, we found the bilateral frontal (Fp1, F8) and parietal-occipital (P7, P3, P8, PO4, O1, and O2) channels activity contributed most to the classification task. The revealed channels, which contributed most to the four-class classification task, coincide with the previous resting and task-related EEG studies. For example, by studying the averaged power spectra between schizophrenia patients, Etevenon et al. (22) reported alpha peak and the mean RMS amplitude is higher over P3-O1 than over P4-O2 for the residual-type of schizophrenic patients, when compared to his matched control sub-group of high-alpha subjects, which presented almost symmetrical occipital alpha peaks and RMS amplitudes. Wix-Ramos et al. (23) found that the mean frequency is higher at Fp1 and Fp2 in bipolar disorder patients than in the control group.

There exist several potential extensions of the present study. First, to comprehensively understand the psychiatric spectrum, samples can be further accumulated to form a larger database that includes various disorders such as Attention-Deficit/Hyperactivity Disorder (ADHD). Second, our sample needs to be further expanded to establish a large database to include more kinds of diseases for classification. Second, to further enhance the performance of the MCRNN, the demographic information (e.g., age, gender) should be considered.

In summary, the deep learning based MCRNN provides a novel solution for identifying psychiatric disorders based on resting-state EEG. By combining with the visualization techniques, the intrinsic relationships among psychiatric disorders are revealed, providing a new perspective for understanding the comorbidity and heterogeneity of psychiatric disorders.

## Data availability statement

The raw data supporting the conclusions of this article will be made available by the authors, without undue reservation.

## Ethics statement

The studies involving human participants were reviewed and approved by the ethics committee of the institutional review board of Second Affiliated Hospital, Zhejiang University School of Medicine. Written informed consent to participate in this study was provided by the participants’ legal guardian/next of kin.

## Author contributions

The current research was designed by WY, LY, ZL, JS, and VC. Acquisition of data was performed by WY and DL. The data were analyzed by WY and LY. WY, LY, JS, and VC wrote the paper. All authors contributed to the article and approved the submitted version.

## Funding

This work was supported by the Chinese National Science Foundation (No. 82022035, 61773380, and 82090031), National Institutes of Health (NIH) grant R01MH118695 and R01MH117107, and National Science Foundation (NSF) grant 2112455.

## Conflict of interest

The authors declare that the research was conducted in the absence of any commercial or financial relationships that could be construed as a potential conflict of interest.

## Publisher’s note

All claims expressed in this article are solely those of the authors and do not necessarily represent those of their affiliated organizations, or those of the publisher, the editors and the reviewers. Any product that may be evaluated in this article, or claim that may be made by its manufacturer, is not guaranteed or endorsed by the publisher.

## Footnotes

^1^ https://sccn.ucsd.edu/eeglab/index.php

^2^ https://www.tensorflow.org/

## Abbreviations

SZ: Schizophrenia
BP: Bipolar disorder
MDD: Major depression disorder
NC: Normal controls
BRMS: Bech-Rafaelsen Mania rating scale
HDRS: Hamilton depression rating scale
PANSS: Positive and negative syndrome scale
rsEEG: Resting-state electroencephalography
sMRI: Structural magnetic resonance imaging
GRU: Gated recurrent unit
ROC: Receiver operating characteristic
UMAP: Uniform manifold approximation and projection
