# Supplementary material for: Multi-scale convolutional recurrent neural network for psychiatric disorder identification in resting-state EEG
Source: Front Psychiatry. 2023 Jun 27;14:1202049. doi: 10.3389/fpsyt.2023.1202049 (PMC10333510; doi:10.3389/fpsyt.2023.1202049)
Supplement: Supplementary file 1 [file Data_Sheet_1.docx]

**Supplementary Materials**

**
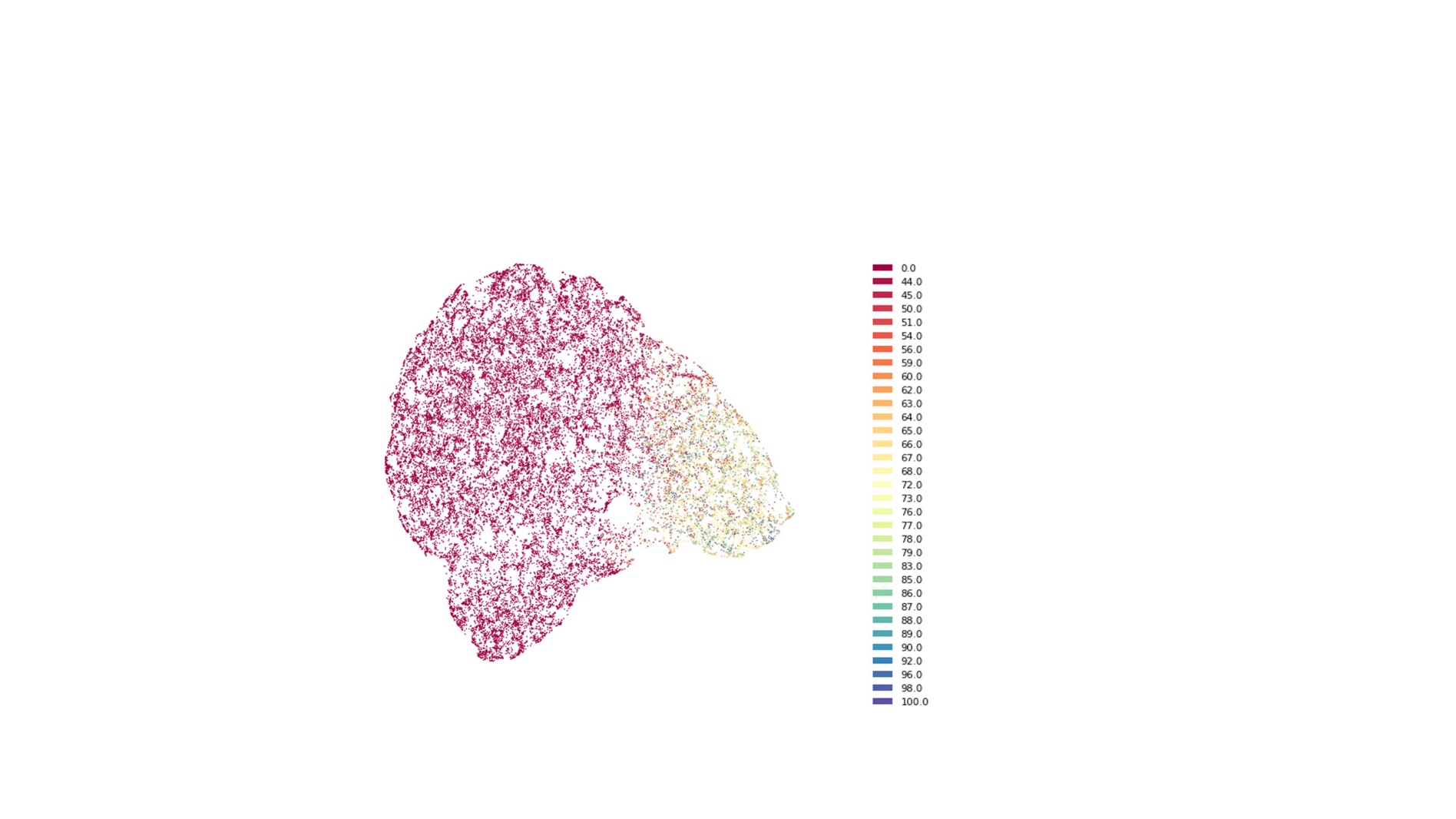
**

**Figure S1.** The PANSS score gradient of schizophrenia patients. The results show that the PNASS score increases when the dots are closer to the schizophrenia pole.

**Table S1. Details of the EEG electrodes and network**

| **Electrode** | **Network** | **Electrode** | **Network** | **Electrode** | **Network** | **Electrode** | **Network** |
| --- | --- | --- | --- | --- | --- | --- | --- |
| **'Fp1'** | Frontal | **'C4'** | Central | **'AF3'** | Frontal | **'P5'** | Parietal |
| **'Fpz'** | None | **'T8'** | Temporal | **'AF4'** | Frontal | **'P1'** | Parietal |
| **'Fp2'** | Frontal | **'M2'** | None | **'AF8'** | Frontal | **'P2'** | Parietal |
| **'F7'** | Frontal | **'CP5'** | Central | **'F5'** | Frontal | **'P6'** | Parietal |
| **'F3'** | Frontal | **'CP1'** | Central | **'F1'** | Frontal | **'PO5'** | Occipital |
| **'Fz'** | None | **'CP2'** | Central | **'F2'** | Frontal | **'PO3'** | Occipital |
| **'F4'** | Frontal | **'CP6'** | Central | **'F6'** | Frontal | **'PO4'** | Occipital |
| **'F8'** | Frontal | **'P7'** | Parietal | **'FC3'** | Central | **'PO6'** | Occipital |
| **'FC5'** | Central | **'P3'** | Parietal | **'FCz'** | None | **'FT7'** | Temporal |
| **'FC1'** | Central | **'Pz'** | None | **'FC4'** | Central | **'FT8'** | Temporal |
| **'FC2'** | Central | **'P4'** | Parietal | **'C5'** | Central | **'TP7'** | Temporal |
| **'FC6'** | Central | **'P8'** | Parietal | **'C1'** | Central | **'TP8'** | Temporal |
| **'M1'** | None | **'POz'** | None | **'C2'** | Central | **'PO7'** | Occipital |
| **'T7'** | Temporal | **'O1'** | Occipital | **'C6'** | Central | **'PO8'** | Occipital |
| **'C3'** | Central | **'O2'** | Occipital | **'CP3'** | Central | **'Oz'** | None |
| **'Cz'** | None | **'AF7'** | Frontal | **'CP4'** | Central |  |  |
